# Supplementary material for: Heteroaryl-Ethylenes as New Lead Compounds in the Fight against High Priority Bacterial Strains
Source: Antibiotics (Basel). 2021 Aug 25;10(9):1034. doi: 10.3390/antibiotics10091034 (PMC8466554; doi:10.3390/antibiotics10091034)
Supplement: Supplementary file 1 [file antibiotics-10-01034-s001.zip › antibiotics-1338436-supplementary.pdf]

antibiotics

MDPI

Article

Heteroaryl-Ethylenes as New Lead Compounds in the Fight Against High Priority Bacterial Strains

Dafne Bongiorno [1,*,†], Nicolò Musso [1,†], Paolo G. Bonacci [2], Dalida A. Bivona [1], Mariacristina Massimino [1], Stefano Stracquadanio [1], Carmela Bonaccorso [2,*], Cosimo G. Fortuna [2] and Stefania Stefani [1]

[1] Department of Biomedical and Biotechnological Sciences (BIMETEC), Section of Microbiology, University of Catania, 95123 Catania, Italy; nmusso@unict.it (N.M.); dalidabivona@gmail.com (D.A.B.); m.cri1503@gmail.com (M.M); s.stracquadanio@hotmail.it (S.S.); stefanis@unict.it (S.S.)
[2] Department of Chemical Sciences, University of Catania, 95125 Catania, Italy; paolo.g.bonacci@gmail.com (P.G.B.); cg.fortuna@unict.it (C.G.F.)
* Correspondence: d.bongiorno@unict.it (D.B.); bonaccorsoc@gmail.com (C.B.)
† These authors contributed equally to this work

Supplementary Materials

Table S1. Structures of the 40 compounds used to generate the QSARmodel in VolSurf+	S2
VolSurf+ computational procedure	S10
Scheme S1. Structures of the investigated PBn compounds	S11
Synthesis of PB7	S12
Synthesis of PB8	S13
Figure S1. ^{1}H-NMR spectrum of PB7	S14
Figure S2. ^{13}C-NMR spectrum of PB7	S15
Figure S3. ^{1}H-NMR spectrum of PB8	S16
Figure S4. ^{13}C-NMR spectrum of PB8	S17
Table S2. IC50 values obtained from the MTT assays after 24 hours of incubation relative to untreated controls.	S18
Table S3. MIC values for ATCC12598 and ATCC51299 with different *inocula*	S19
Table S4. PB4 MIC values for ATCC 17978 and ATCC25922	S20
with different *inocula*	S21
References	

Table S1. Structures of the 40 compounds used to generate the QSARmodel in VolSurf+.

**Molecular Structure**<br>**SMILE**	**ID VS+**	**MIC**<br>**(μg/mL)**	**[Ref]**
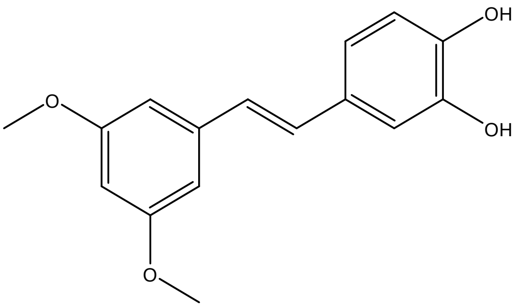<br>Oc1ccc(/C=C/c2cc(OC)cc(OC)c2)cc1O	[33]-1	128	[1]
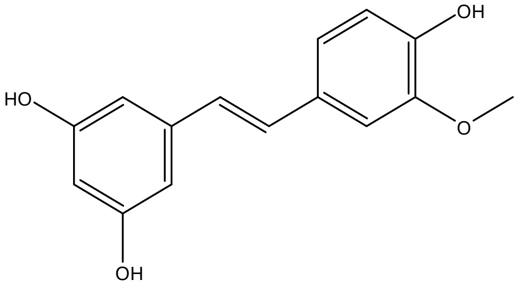<br>Oc1ccc(cc1OC)/C=C/c1cc(O)cc(O)c1	[33]-2	256	[1]
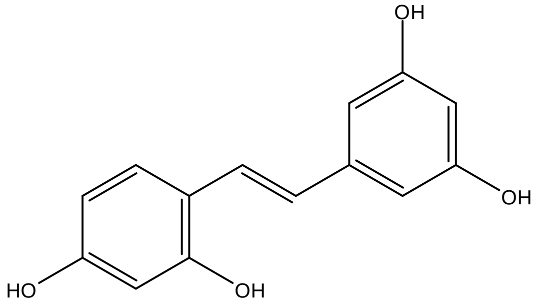<br>Oc1cc(O)ccc1/C=C/c1cc(O)cc(O)c1	[33]-3	256	[1]
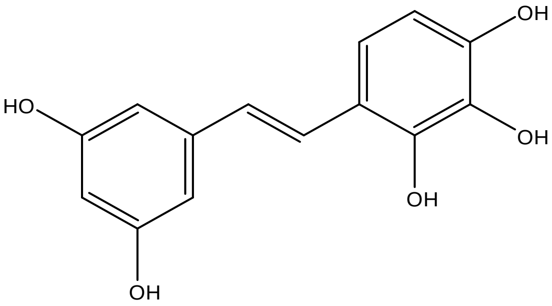<br>Oc1ccc(/C=C/c2cc(O)cc(O)c2)c(O)c1O	[33]-4	64	[1]

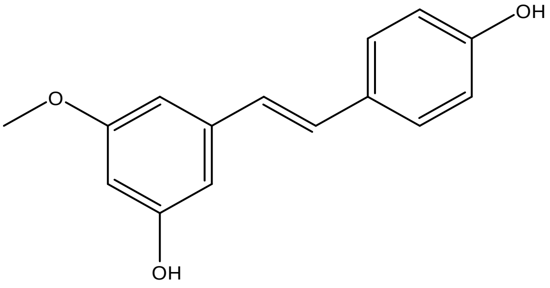 Oc1cc(cc(OC)c1)/C=C/c1ccc(O)cc1	[33]-5	128	[1]
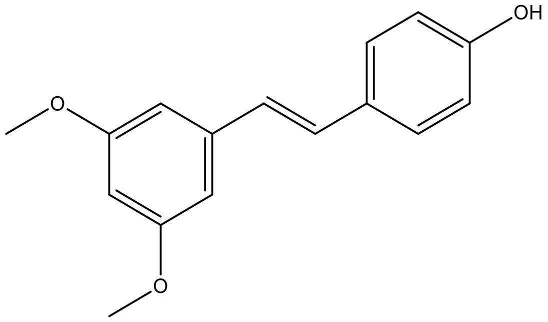 COc1cc(OC)cc(\C=C\c2ccc(O)cc2)c1	[33]-6	32	[1]
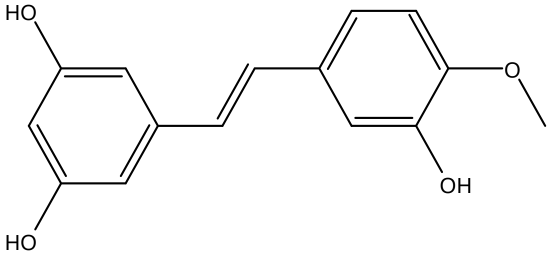 Oc1cc(cc(OC)c1)/C=C/c1ccc(O)cc1	[33]-8	256	[1]
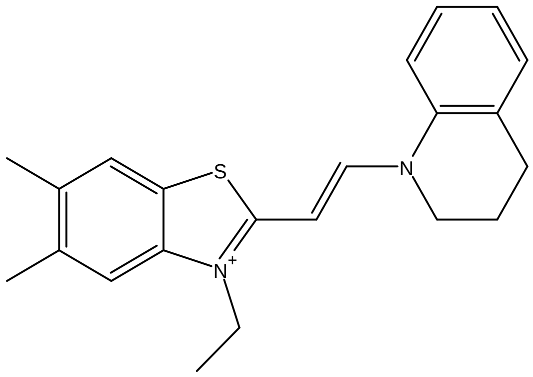 Cc1cc2c(cc1C)sc(/C=C/N1CCCc3ccccc31)[n+]2CC	[NE]-1	4	[2]

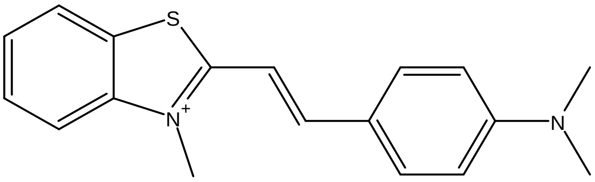 CN(C)c1ccc(cc1)/C=C/c1sc2ccccc2[n+]1C	[BI]-1	8	[3]
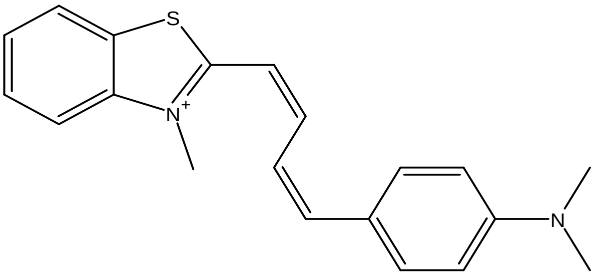 CN(C)c1ccc(cc1)/C=C\C=C/c1sc2ccccc2[n+]1C	BI-2	4	[3]
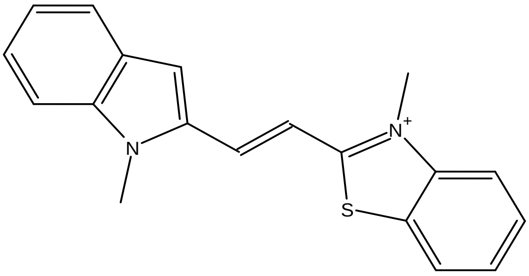 Cn1c2ccccc2cc1/C=C/c1sc2ccccc2[n+]1C	BI-3	4	[3]
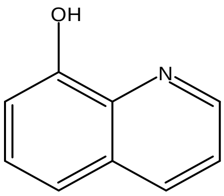 Oc1cccc2cccnc12	CH-1	6.89	[3]
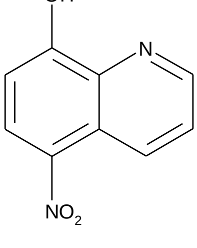 Oc1ccc(c2cccnc12)[N+](=O)[O-]	CH-2	42.07	[3]

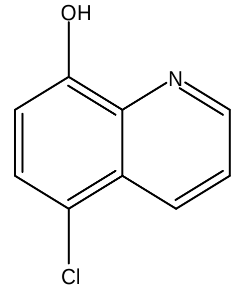 Oc1ccc(Cl)c2cccnc12	CH-3	89.1	[3]
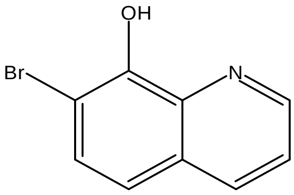 Oc1c(Br)ccc2cccnc12	CH-4	35.71	[3]
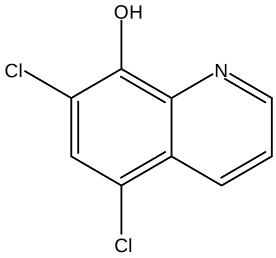 Oc1c(Cl)cc(Cl)c2cccnc12	CH-5	37.37	[3]
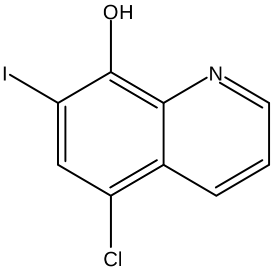 Oc1c(I)cc(Cl)c2cccnc12	CH-6	26.19	[3]

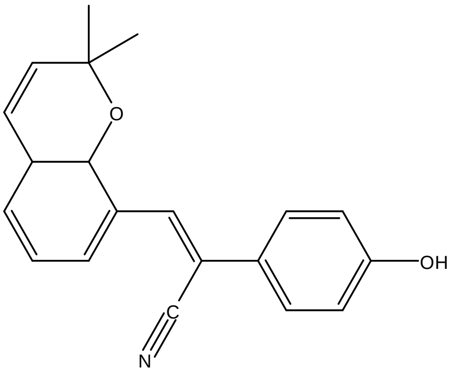 Oc1ccc(cc1)C(\C#N)=C\C1=CC=CC2C=CC(C)(C)OC12	CN-1	3	[4]
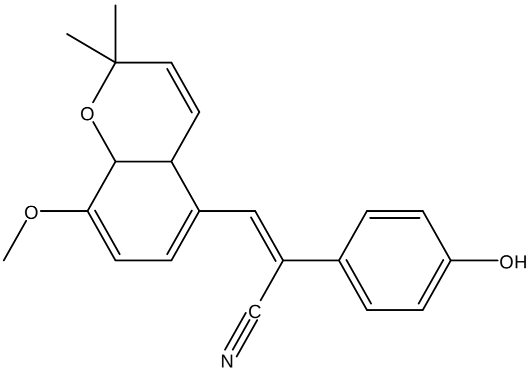 Oc1ccc(cc1)C(\C#N)=C\C1=CC=C(OC)C2OC(C)(C)C=CC12	CN-2	12	[4]
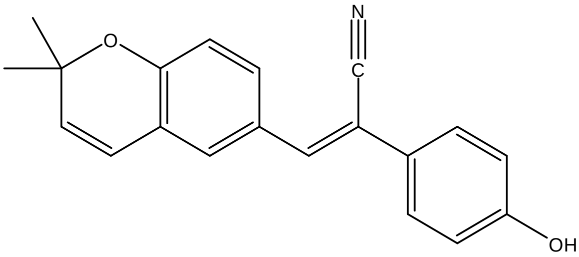 Oc1ccc(cc1)C(\C#N)=C\c1ccc2OC(C)(C)C=Cc2c1	CN-3	25	[4]
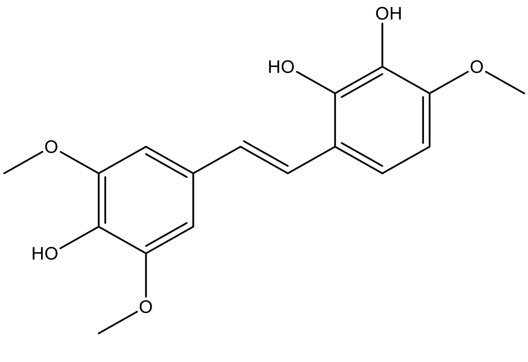	CW	16	[5]

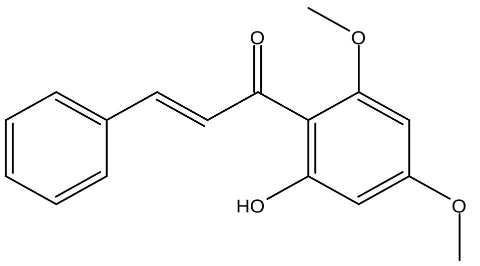 COc1cc(cc(O)c1C(=O)/C=C/c1ccccc1)OC	EL-1	40	[6]
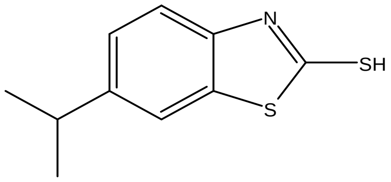 CC(C)c1ccc2nc(S)sc2c1	FR-2a	25	[7]
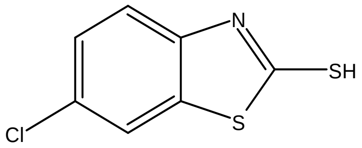 Clc1ccc2nc(S)sc2c1	FR-2b	12.5	[7]
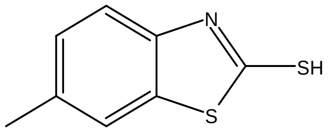 Cc1ccc2nc(S)sc2c1	FR-2c	50	[7]
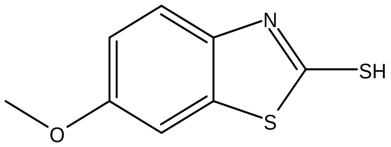 COc1ccc2nc(S)sc2c1	FR-2d	50	[7]
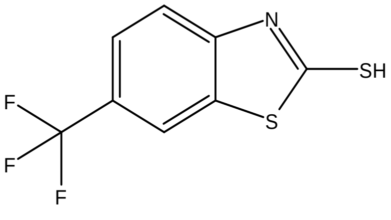 FC(F)(F)c1ccc2nc(S)sc2c1	FR-2e	3.12	[7]

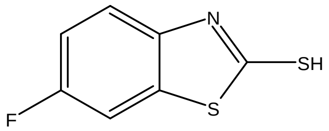<br>Fc1ccc2nc(S)sc2c1	FR-2f	25	[7]
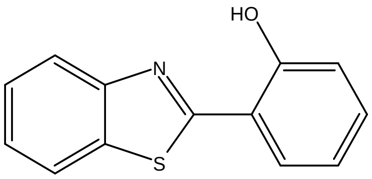<br>Oc1ccccc1c1nc2ccccc2s1	RL-5a	256	[8]
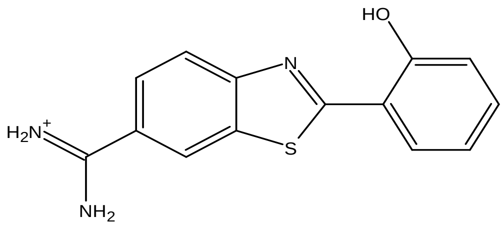<br>[NH2+]=C(N)c1ccc2nc(sc2c1)c1ccccc1O	RL-5d	32	[8]
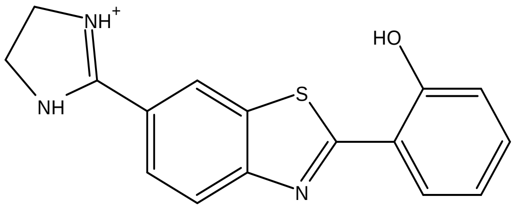<br>Oc1ccccc1c1nc2ccc(cc2s1)C1=[NH+]CCN1	RL-5e	256	[8]
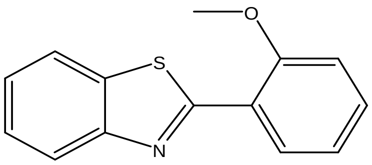<br>COc1ccccc1c1nc2ccccc2s1	RL-6a	256	[8]
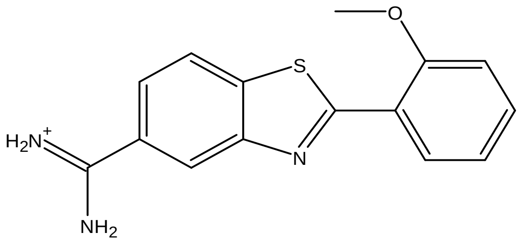<br>[NH2+]=C(N)c1cc2nc(sc2cc1)c1ccccc1OC	RL-6d	128	[8]

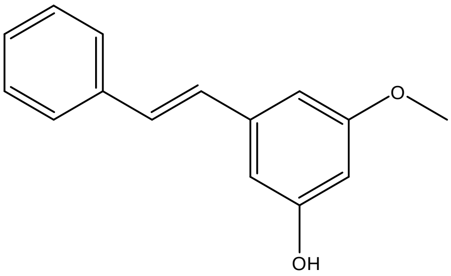 Oc1cc(cc(OC)c1)/C=C/c1ccccc1	SC-1	16	[9]
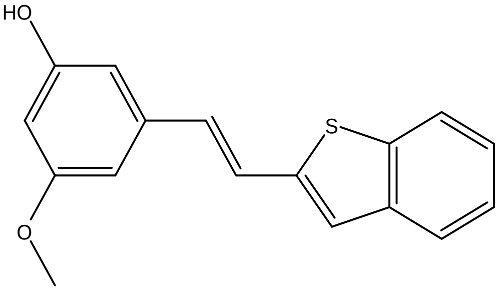 Oc1cc(cc(OC)c1)/C=C/c1cc2ccccc2s1	SK-03-92	2	[10]
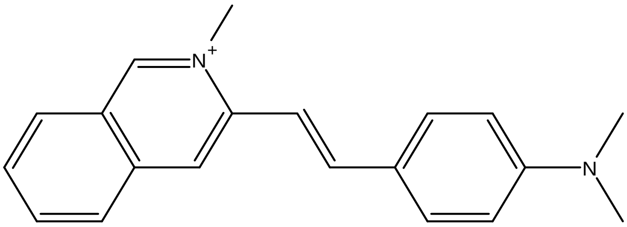 CN(C)c1ccc(cc1)/C=C/c1cc2ccccc2c[n+]1C	Q-2	18.75	[11]
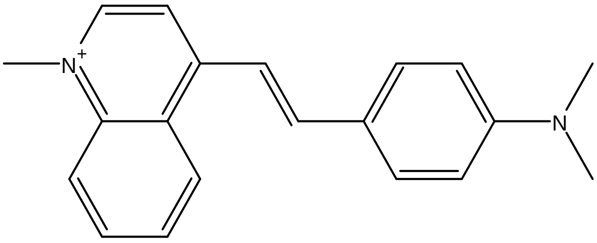 CN(C)c1ccc(cc1)/C=C/c1cc[n+](C)c2ccccc21	Q-3	4.7	[11]

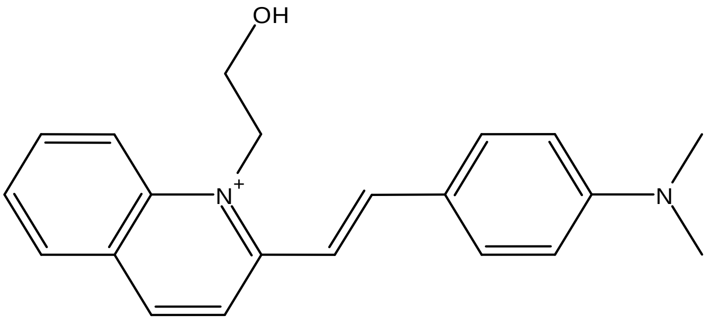 CN(C)c1ccc(cc1)/C=C/c1ccc2ccccc2[n+]1CCO	Q-5	75	[11]
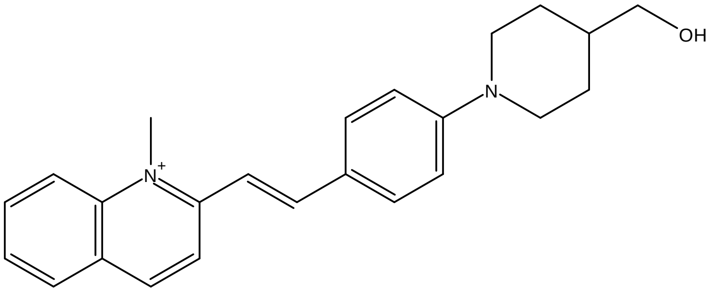 C[n+]1c2ccccc2ccc1/C=C/c1ccc(cc1)N1CCC(CC1)CO	Q-7	75	[11]
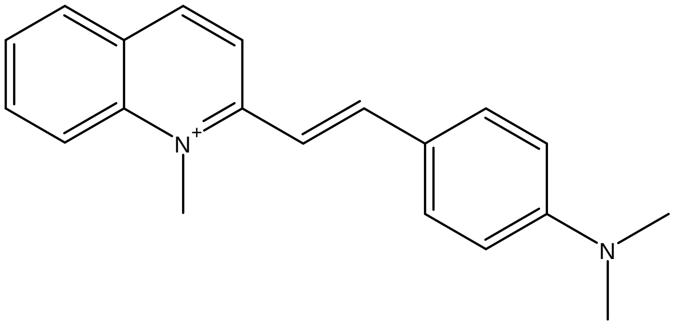 CN(C)c1ccc(cc1)/C=C/c1ccc2ccccc2[n+]1C	Q-11	4.7	[11]

VolSurf+ **computational procedure.**

VolSurf+ is a computational procedure designed to produce and to explore the physicochemical property space of a molecule starting from 3D interaction energy maps. The basic concept of VolSurf+ is to compress the information present in 3D maps into a few quantitative numerical descriptors that are very simple to understand and to interpret. VolSurf+ does this by selecting the most appropriate descriptors and parameterization according to the type of the 3D map under study.

Interaction fields with a water probe (OH2), a hydrophobic probe (DRY) plus an H-bond donor (NH) and an H-bond acceptor (=O) probes are calculated all around the target molecules as in the program GRID. The information contained in the MIF is transformed into a quantitative scale by calculating the volume or the surface of the interaction contours.

The VolSurf+ procedure is as follows: i) in the first step, the 3D molecular field is generated from the interactions of the OH2, the DRY, O and N1 probe around a target molecule; ii) the second step consists in the calculation of descriptors from the 3D maps obtained in the first step. The molecular descriptors obtained, called VolSurf+ descriptors, refer to molecular size and shape, to hydrophilic and hydrophobic regions and to the balance between them, to molecular diffusion, LogP, LogD, to the "charge state" descriptors, to the 3D pharmacophoric descriptors and to some descriptors on some rilevant ADME properties.[12–14]

Finally, the Partial Least Squares (PLS) chemometric tools [15] is used to create relationships of the VolSurf+ descriptor matrix with the desired biological properties. The scheme of the VolSurf+ programme steps and a detailed definition of Volsurf+ descriptors have been reported.[16]

Scheme S1. Structures of the investigated PBn compounds.

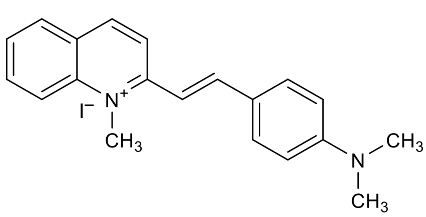

PB1

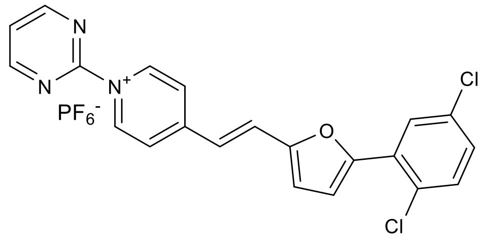

PB5

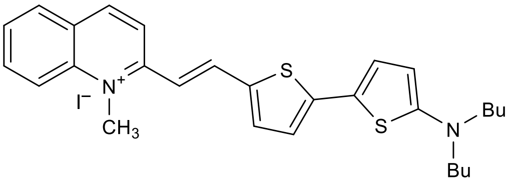

PB2

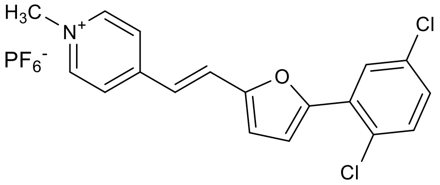

PB6

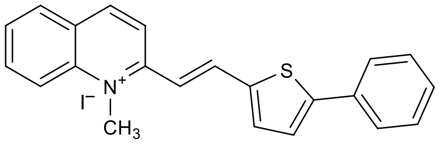

PB3

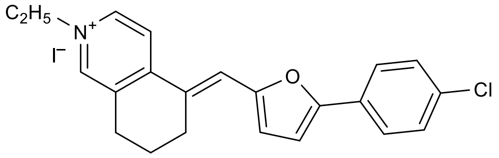

PB7

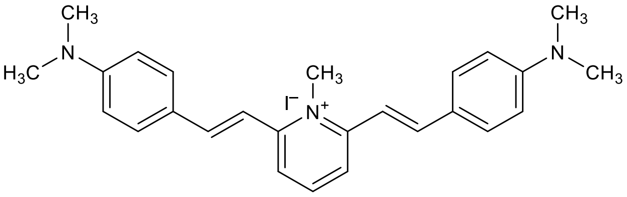

PB4

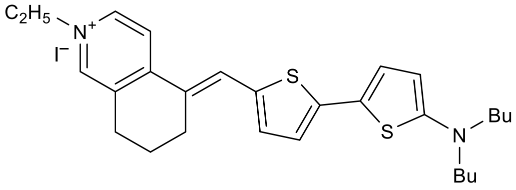

PB8

Synthesis of PB7

2-ethyl-5,6,7,8-tetrahydroisoquinolin-2-ium iodide (70.1 mg, 0.24 mmol) and 5-(3-chlorophenyl) furan-2-carbaldehyde (55.8 mg, 0.27 mmol) were mixed in 2 mL of ethanol and a minimum amount of dichloromethane to ensure solubilization of the aldehyde. A catalytic amount of Piperidine (20 μL) was added to the mixture and the reaction was carried out under reflux and vigorous stirring. The reaction was monitored through TLC and stopped after 17 hours.

The solvent was removed *in vacuo* and the solid purified through precipitation (dichloromethane / ethyl ether). The identity of the isolated product was confirmed by NMR spectroscopy. The yield of the synthesis and purification process is 42.2%.

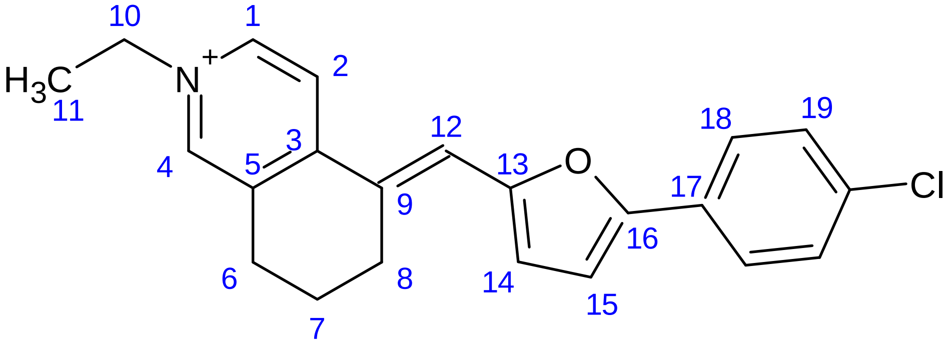

^{1}H-NMR (DMSO, 500 MHz, 300 K); δ(ppm): 8.82 (s; 1H; H4); 8.73 (d; 1H; J= 14 Hz; H1); 8.42 (d; 1H; J=14 Hz; H2); 7.83 (d; 2H; J= 18 Hz; H18); 7.69 (s; 1H; H12); 7.56 (d; 2H; J=18 Hz; H19); 7.3 (d; 1H; J=7 Hz; H14); 7.08 (d; 1H; J = 7 Hz; H15); 4.60 (q; 2H; J= 14 Hz; H10); 3.04 (t; 2H; J=12 Hz; H8); 2.88 (t; 2H; J=12 Hz; H6); 1.91 (q; 2H; J=12 Hz; H7); 1.52 (t; 3H; H= 14 HZ; H11).

^{13}C-NMR (DMSO, 125 MHz, 300 K); δ(ppm): 154.60, 152.45, 151.41, 143.44, 141.11, 136.95, 133.42, 129.69, 129.18, 128.61, 126.18, 121.69, 121.57, 119.76, 110.40, 55.39, 27.61, 27.10, 21.08, 16.59.

Synthesis of PB8

2-ethyl-5,6,7,8-tetrahydroisoquinolin-2-ium iodide (72.0 mg, 0.25 mmol) and 5′-(dibuthylamino)[2,2′-bithiophene]-5-carbaldehyde (88 mg, 0.27 mmol) were mixed in 2 mL of ethanol and a minimum amount of dichloromethane to ensure solubilization of the aldehyde. A catalytic amount of Piperidine (20 μL) was added to the mixture and the reaction was carried out under reflux and vigorous stirring. The reaction was monitored through TLC and stopped after 17 hours. The solvent was removed *in vacuo* and the oily product purified through chromatographic column (gradient elution, dichloromethane - dichloromethane: methanol = 90 : 10). The identity of the isolated product was confirmed by NMR spectroscopy. The yield of the synthesis and purification process is 47.3%.

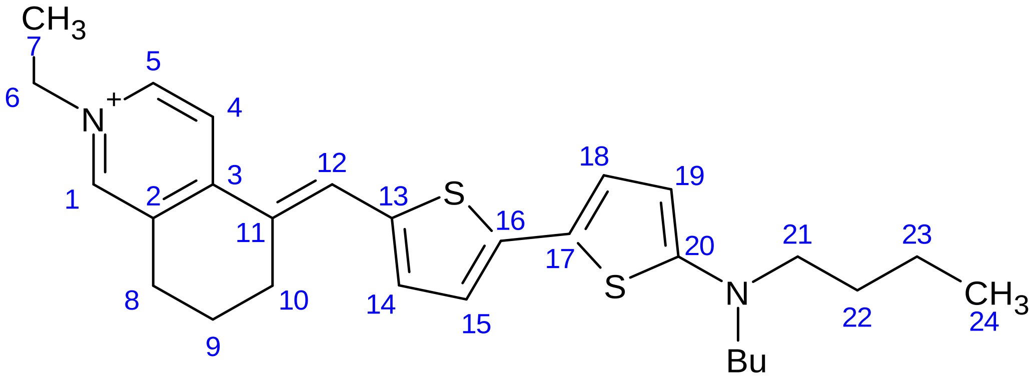

^{1}H-NMR (DMSO, 500 MHz, 300 K); δ(ppm): 8.87 (s; 1H; H1); 8.77 (d; 1H; J=13.0 Hz; H4); 8.13 (d; 1H; J=14.0 Hz; H5); 7.73 (s; 1H; H12); 7.35 (d; 1H; H19); 7.03 (d; 1H; H18); 6.95 (d; 1H; H15); 5.75 (d; 1H; H14); 4.7 (d; 2H; H6); 3.27 (t; 3H; H7); 2.95 (t; 2H; H8); 2.87 (m; 2H; H9); 2.0 (d; 2H; J=11.0 Hz; H10); 1.63 (m; 12H; J=14.0 Hz; H21+H22+ H23); 0.97 (t; 6H; J=14 Hz; H24).

^{13}C-NMR (DMSO, 125 MHz, 300 K); δ(ppm): 159.12, 152.84, 146.55, 141.59, 139.96, 136.32, 136.07, 134.40, 130.14, 126.37, 124.69, 120.57, 120.48, 118.02, 101.66, 55.39, 53.40, 29.19, 27.58, 27.40, 20.87, 20.25, 16.90, 13.90.

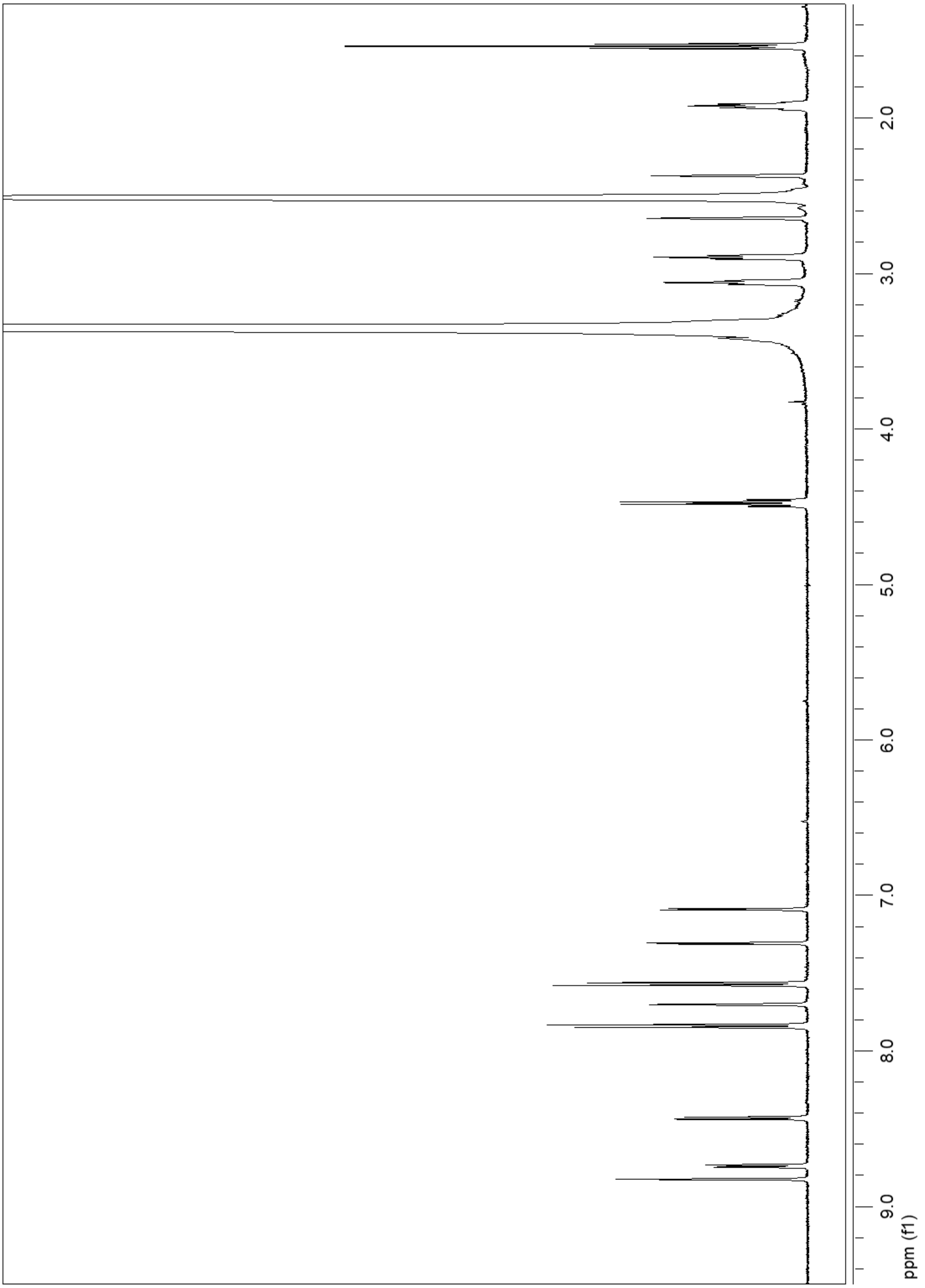

Figure S1. ^{1}H-NMR spectrum of **PB7** (500 MHz, 300 K, DMSO-D6).

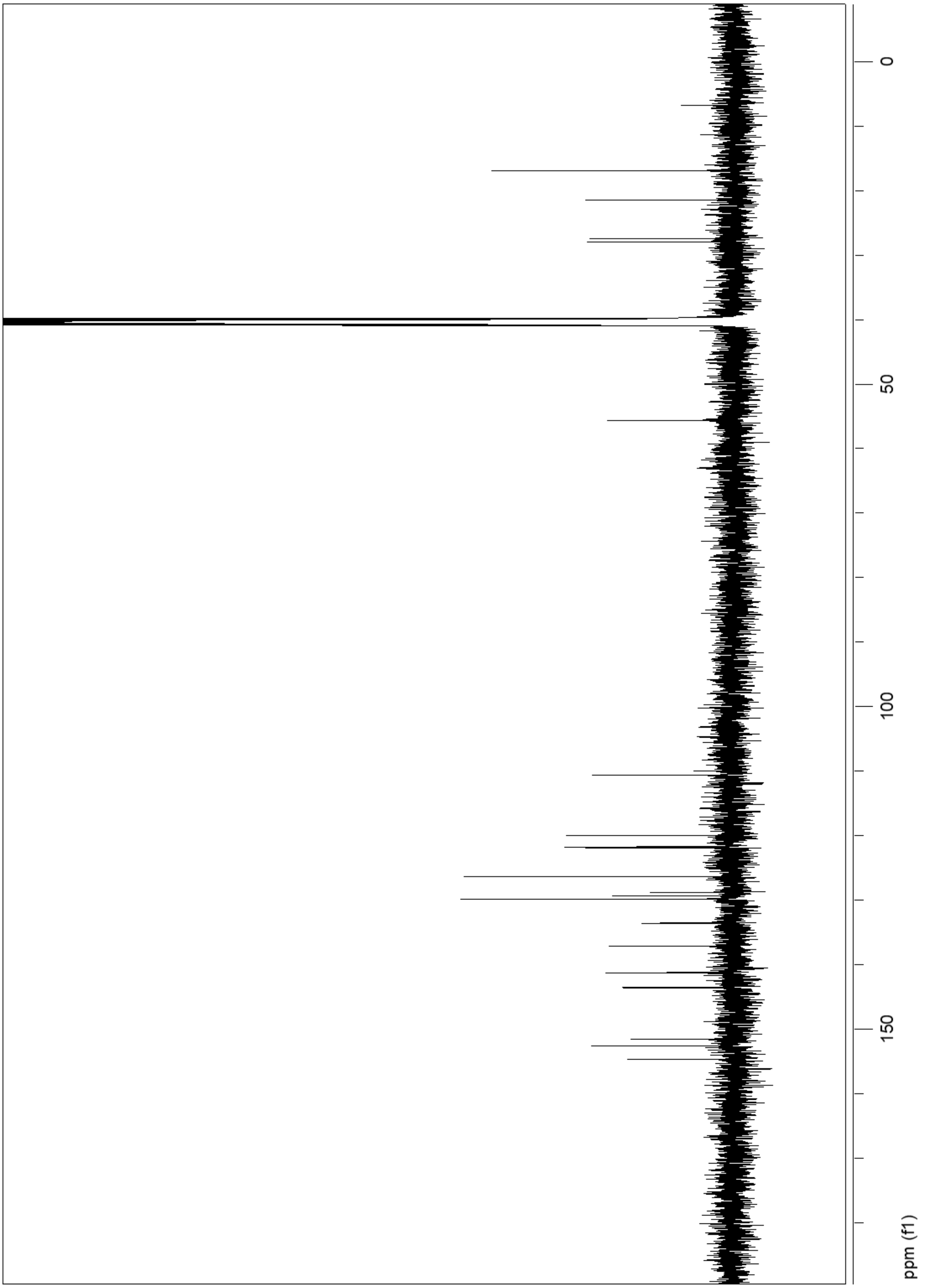

Figure S2. ^{13}C-NMR spectrum of **PB7** (125 MHz, 300 K, DMSO-D6).

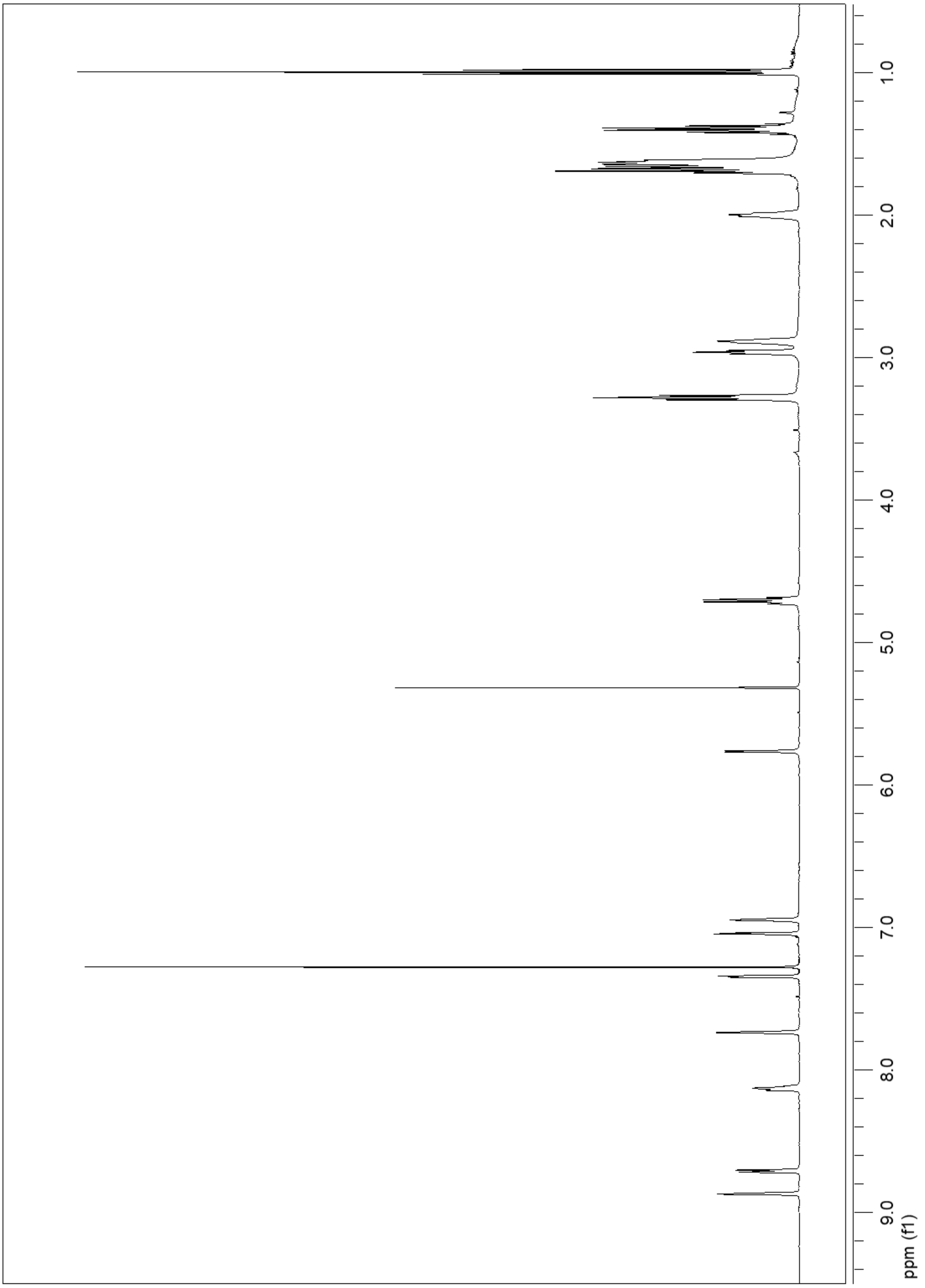

Figure S3. ^{1}H-NMR spectrum of **PB8** (500 MHz, 300 K, DMSO-D6).

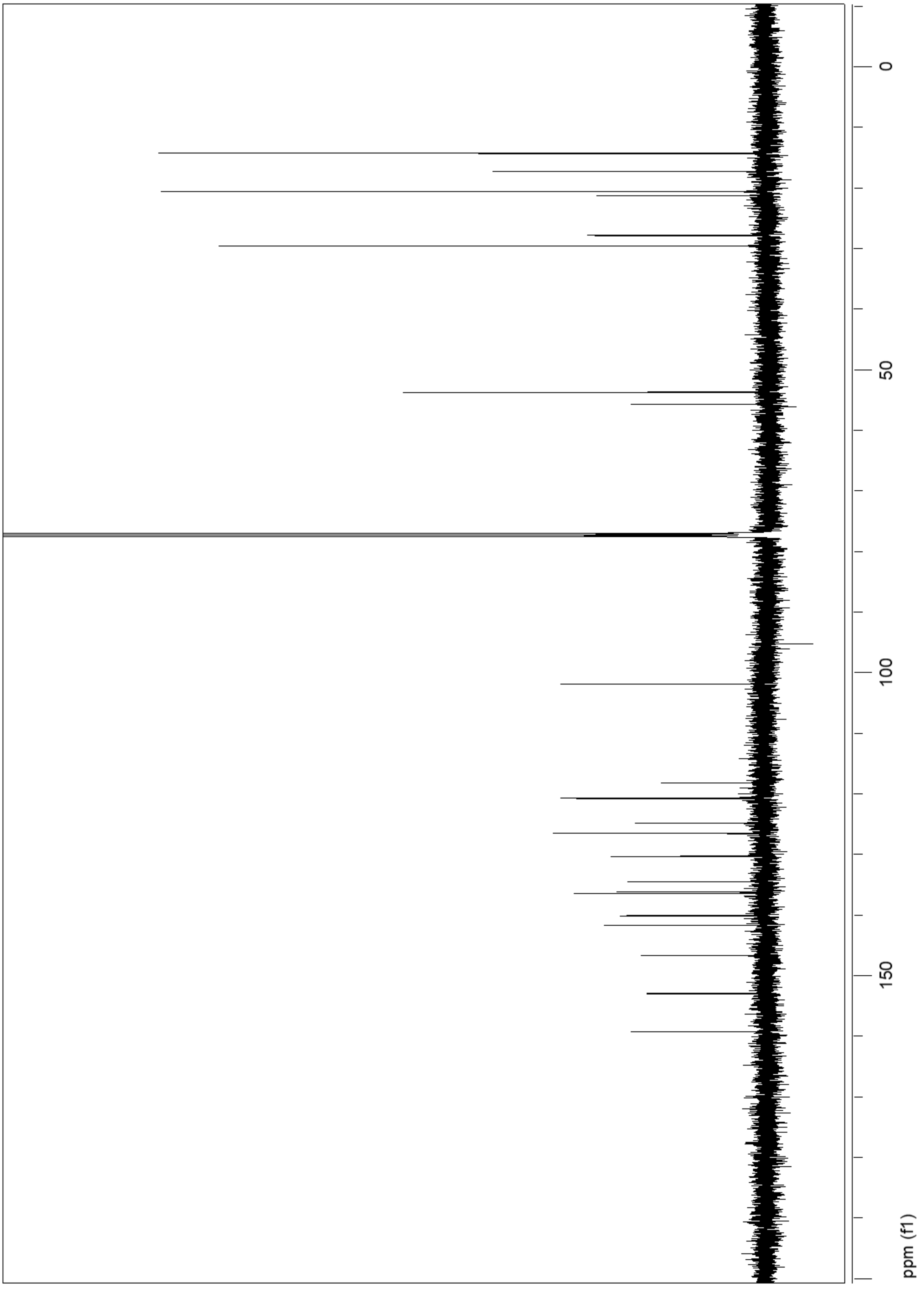

Figure S4. ^{13}C-NMR spectrum of **PB8** (125 MHz, 300 K, DMSO-D6).

Table S2. IC50 values obtained from the MTT assays after 24 hours of incubation relative to untreated controls.

**Compounds.**	**IC50 (μM)**	**95% confidence interval (μM)**
PB1	1.202	0.6061 to 2.384
PB2	0.6025	0.2783 to 1.305
PB3	0.871	0.3319 to 2.286
PB4	0.3186	0.09087 to 1.117
PB5	3.901	1.566 to 9.717
PB6	6.788	1.849 to 24.92
PB7	1.362	0.5308 to 3.488
PB8	5.013	1.488 to 17.10
5-FU	27.361	10.96 to 87.70

Table S3. MIC values for ATCC12598 and ATCC51299 with different *inocula.*

		MIC mg/L [µM]			
		**PB4**	**PB5**	**PB7**	**PB8**
***S. aureus* ATCC12598**	1.00×10^{7}	4 [7.82]	8 [14.81]	32 [69.00]	4 [6.91]
	1.00×10^{6}	0.25 [0.49]	4 [7.40]	8 [17.25]	2 [3.46]
	1.00×10^{5}	0.25 [0.49]	4 [7.40]	8 [17.25]	2 [3.46]
	1.00×10^{4}	0.03 [0.06]	0.06 [0.12]	4 [8.63]	2 [3.46]
	1.00×10^{3}	<0.015 [<0.03]	0.03 [0.06]	4 [8.63]	2 [3.46]
	1.00×10^{2}	<0.015 [<0.03]	0.03 [0.06]	4 [8.63]	2 [3.46]
	1.00×10^{1}	<0.015 [<0.03]	0.03 [0.06]	2 [4.31]	0.5 [0.86]
***E. faecalis* ATCC51299**	1.00×10^{7}	0.5 [0.98]	4 [7.40]	32 [69.00]	32 [55.30]
	1.00×10^{6}	0.5 [0.98]	4 [7.40]	16 [34.50]	8 [13.83]
	1.00×10^{5}	0.5 [0.98]	4 [7.40]	8 [17.25]	4 [6.91]
	1.00×10^{4}	0.12 [0.24]	1 [1.85]	8 [17.25]	4 [6.91]
	1.00×10^{3}	0.12 [0.98]	1 [1.85]	8 [17.25]	4 [6.91]
	1.00×10^{2}	0.25 [0.29]	1 [1.85]	8 [17.25]	4 [6.91]
	1.00×10^{1}	0.06 [0.12]	0.5 [0.93]	8 [17.25]	0.5 [0.86]

Table S4. PB4 MIC values for ATCC 17978 and ATCC25922 with different *inocula.*

	**MIC mg/L [μM]**	
		**PB4**
***A. baumannii* ATCC 17978**	1.00×10⁷	1 [1.96]
	1.00×10⁶	0,25 [0.49]
	1.00×10⁵	0.25 [0.49]
	1.00×10⁴	0.25 [0.49]
	1.00×10³	<0.25 [<0.49]
	1.00×10²	<0.25 [<0.49]
	1.00×10¹	<0.25 [<0.49]
***E. coli* ATCC25922**	1.00×10⁷	2 [3.91]
	1.00×10⁶	1 [1.96]
	1.00×10⁵	1 [1.96]
	1.00×10⁴	0.5 [0.98]
	1.00×10³	0.5 [0.98]
	1.00×10²	0.5 [0.98]
	1.00×10¹	0.5 [0.98]

References

1. Zakova, T.; Rondevaldova, J.; Bernardos, A.; Landa, P.; Kokoska, L. The relationship between structure and in vitro antistaphylococcal effect of plant-derived stilbenes. *Acta Microbiol. Immunol. Hung.* **2018**, *65*, 467–476.
2. Eibergen, N.R.; Im, I.; Patel, N.Y.; Hergenrother, P.J. Identification of a novel protein synthesis inhibitor active against Gram-positive bacteria. **2012**, *61801*, 574–583.
3. Chauhan, J.; Yu, W.; Cardinale, S.; Opperman, T.J.; MacKerell Jr, A.D.; Fletcher, S.; de Leeuw, E.P.H. Optimization of a benzothiazole indolene scaffold targeting bacterial cell wall assembly. *Drug Des. Devel. Ther.* **2020**, *14*, 567–574.
4. Nicolaou, K.C.; Roecker, A.J.; Barluenga, S.; Pfefferkorn, J.A.; Cao, G.Q. Discovery of novel antibacterial agents active against methicillin-resistant Staphylococcus aureus from combinatorial benzopyran libraries. *ChemBioChem* **2001**, *2*, 460–465.
5. Eloff, J.N.; Katerere, D.R.; Mcgaw, L.J. The biological activity and chemistry of the southern African Combretaceae. *J Ethnopharmacol*. **2008**, *119*, 686–699.
6. Eloff, J.N.; Katerere, D.R.; Mcgaw, L.J. The biological activity and chemistry of the southern African Combretaceae. **2008**, *119*, 686–699.
7. Franchini, C.; Muraglia, M.; Corbo, F.; Florio, M.A.; Di Mola, A.; Rosato, A.; Matucci, R.; Nesi, M.; Van Bambeke, F.; Vitali, C. Synthesis and biological evaluation of 2-mercapto-1,3-benzothiazole derivatives with potential antimicrobial activity. *Arch. Pharm. (Weinheim).* **2009**, *342*, 605–613.
8. Racané, L.; Ptiček, L.; Fajdetić, G.; Tralić-kulenović, V.; Klobučar, M.; Starčević, K. Bioorganic Chemistry Green synthesis and biological evaluation of 6-substituted-2- (2-hydroxy / methoxy phenyl) benzothiazole derivatives as potential antioxidant , antibacterial and antitumor agents. *Bioorg. Chem.* **2020**, *95*, 103537.
9. Kabir, M.S.; Engelbrecht, K.; Polanowski, R.; Krueger, S.M.; Ignasiak, R.; Rott, M.; Schwan, W.R.; Stemper, M.E.; Reed, K.D.;

Sherman, D.; et al. New classes of Gram-positive selective antibacterials: Inhibitors of MRSA and surrogates of the causative agents of anthrax and tuberculosis. *Bioorganic Med. Chem. Lett.* **2008**, *18*, 5745–5749.
10. Schwan, W.R.; Kabir, M.S.; Kallaus, M.; Krueger, S.; Monte, A.; Cook, J.M. Synthesis and minimum inhibitory concentrations of SK-03-92 against Staphylococcus aureus and other gram-positive bacteria. *J. Infect. Chemother.* **2012**, *18*, 124–126.
11. Kim, E.; Lee, S.H.; Lee, S.J.; Kwon, O.P.; Yoon, H. New antibacterial-core structures based on styryl quinolinium. *Food Sci. Biotechnol.* **2017**, *26*, 521–529.
12. Berellini, G.; Cruciani, G.; Mannhold, R. Pharmacophore, Drug Metabolism, and Pharmacokinetics Models on Non-Peptide AT_1, AT_2, and AT_1/AT_2 Angiotensin II Receptor Antagonists. *J. Med. Chem.* **2005**, *48*, 4389–4399.
13. Crivori, P.; Cruciani, G.; Carrupt, P.-A.; Testa, B. Predicting blood-brain barrier permeation from three-dimensional molecular structure. *J. Med. Chem.* **2000**, *43*, 2204–2216.
14. Carosati, E.; Sciabola, S.; Cruciani, G. Hydrogen Bonding Interactions of Covalently Bonded Fluorine Atoms: From Crystallographic Data to a New Angular Function in the GRID Force Field. *J. Med. Chem.* **2004**, *47*, 5114–5125.
15. WOLD, S.; SJÖSTRÖM, M. SIMCA: A method for analyzing chemical data in terms of similarity and analogy. In *Chemometrics: Theory and Application*; Kowalski, B.R., Ed.; 1977; pp. 243–282.
16. Fortuna, C.G.; Barresi, V.; Musumarra, G. Design, synthesis and biological evaluation of trans 2-(thiophen-2-yl)vinyl heteroaromatic iodides. *Bioorg. Med. Chem.* **2010**, *18*, 4516–4523.
